# Infestation Level Influences Oviposition Site Selection in the Tomato Leafminer *Tuta absoluta* (Lepidoptera: Gelechiidae)

**DOI:** 10.3390/insects5040877

**Published:** 2014-11-11

**Authors:** Thomas Bawin, Lara De Backer, David Dujeu, Pauline Legrand, Rudy Caparros Megido, Frédéric Francis, François J. Verheggen

**Affiliations:** Functional and Evolutionary Entomology, Gembloux Agro-Bio Tech, University of Liege, 2 Passage des Déportés, B-5030 Gembloux, Belgium; E-Mails: Thomas.bawin@doct.ulg.ac.be (T.B.); ldebacker@doct.ulg.ac.be (L.D.B.); ddujeu@student.ulg.ac.be (D.D.); plegrand@alumni.ulg.ac.be (P.L.); r.caparros@doct.ulg.ac.be (R.C.M.); frederic.francis@ulg.ac.be (F.F.)

**Keywords:** *Scrobipalpuloides absoluta*, *Solanum lycopersicum*, insect interaction, attraction, oviposition, volatile organic compound

## Abstract

The tomato leafminer, *Tuta absoluta* (Lepidoptera: Gelechiidae), is a devastating pest that develops principally on solanaceous plants throughout South and Central America and Europe. In this study, we tested the influence of three levels of *T. absoluta* infestations on the attraction and oviposition preference of adult *T. absoluta*. Three infestation levels (*i.e.*, non-infested plants, plants infested with 10 *T. absoluta* larvae, and plants infested with 20 *T. absoluta* larvae) were presented by pairs in a flying tunnel to groups of *T. absoluta* adults. We found no differences in terms of adult attraction for either level of infestations. However, female oviposition choice is influenced by larvae density on tomato plants. We discuss the underlying mechanisms and propose recommendations for further research.

## 1. Introduction

The tomato leafminer, *Tuta absoluta* (Lepidoptera: Gelechiidae), is a widespread invasive species damaging economically important cultivated solanaceous crop plants [1]. It is a serious threat to commercial tomato and potato production in South and Central America as well as in Europe. Larvae feed on the mesophyll of all aerial parts of the plants, as well as on the fruit, resulting in significant yield loss and cosmetic damage to fresh market tomato [1,2,3].

Chemical applications remain the most effective control methods available to reduce *T. absoluta* threat levels. However, the need for alternative control methods is strengthened by the appearance of resistant populations [4] as well as by the side effects of pesticides on beneficial arthropods [5]. No tomato cultivars are entirely resistant to *T. absoluta*, even if some are less susceptible [6]. Preventing pest mating by using synthetic pheromones has been developed. However, the ability of *T. absoluta* females to reproduce parthenogenetically weakened any of these pheromone-based control methods [7]. Biological control approaches using indigenous natural enemies is among the most promising. A list of the Afro-Eurasian natural enemies of *T. absoluta*, including Braconid parasitoids and Mirid predatory bugs, has been compiled, and their potential for inclusion in sustainable control strategies discussed [8]. However, to optimize such effective environmentally sound control strategies, it is necessary to understand the relationships occurring between this leafminer and its host plants [9].

The attraction and oviposition of female *T. absoluta* are mediated by the volatile signature of their host plant [10]. Tomato leaf odors mainly include volatile terpenoid compounds which elicit in mated females upwind orientation flight followed by landing as well as egg-laying. These observations demonstrate the essential role of plant volatiles in *T. absoluta* host-finding behavior. Similar observations were made for potato plants: the volatile headspace of *Solanum tuberosum* is implicated in the host selection by female *T. absoluta* [11]. In the present study, we evaluate the impact of the level of *T. absoluta* infestations on the attraction and oviposition site selection by mated *T. absoluta*.

## 2. Materials and Methods

### 2.1. Plant Material

Tomato (*Solanum lycopersicum* cv. Moneymaker) were cultivated in a greenhouse (25 ± 5 °C) in plastic pots (20 cm diameter × 20 cm height) filled with loam (VP113BIO; Peltracom, Belgium) and grown with a 16L:8D photoperiod. The plants were watered once every 2 days.

### 2.2. Insect Rearing

In July 2011, 200 third instar larvae of the tomato leafminer, *Tuta absoluta*, were collected from a commercial tomato plantation located in Saint-Andiol (France), and were subsequently kept under laboratory conditions at 24 ± 1 °C, 60%–70% RH, and with a 16L:8D photoperiod. *T. absoluta* colony was reared on tomato in net cages (46.5 × 46.5 × 46.5 cm). Caterpillars were provided with fresh plants three times a week until pupation. Five generations were raised before starting any experiment to maintain a 1:1 female/male ratio in the colony and avoid parthenogenetic reproduction of the females (and a consequent decrease in fitness) [7].

### 2.3. Bioassays

Flying tunnels (232.5 × 46.5 × 46.5 cm) were used to evaluate the attraction of *T. absoluta* males and females as well as the oviposition site selection by females facing non-infested tomato plants *versus* *T. absoluta*-infested plants. The tunnels were divided into three non-ventilated areas [11]: a central area for insect release and two areas at opposite sites containing the plants. Three dual-choice assays were conducted: (1) non-infested tomato plants* versus* tomato plants infested with 10 *T. absoluta* larvae; (2) non-infested tomato plants* versus* tomato plants infested with 20 *T. absoluta* larvae; and (3) tomato plants infested with 10 *T. absoluta* larvae* versus* tomato plants infested with 20 larvae. The tomato plants used in the experiments were 4 weeks old and corresponded to a 107 BBCH (Biologische Bundesanstalt, Bundessortenamt und Chemische Industrie) code (approximately 30 cm high and 6–7 leaves). Infestations were performed by cutting, from the rearing colony, 10 or 20 small leaf pieces (depending on the number of individuals per plant required for the experimental setup), each containing a single second *T. absoluta* instar, which were randomly deposited on the tomato plants intended for the experiments. Non-infested and infested tomato plants were kept 4 days under laboratory conditions until the bioassays. After incubation, tomato plants were set in both sides of the flying tunnel, and twenty newly emerged *T. absoluta* adults (<24 h) were placed in the neutral area at a distance of 116.5 cm from each plant. After 48 h, the number of males, females, and eggs laid on each plant were counted. Forty-eight hours allow the couples to explore the tunnel, to mate, and also allow the females to lay eggs in sufficient numbers on one of the two plants. Each plant combination was randomly tested four times (for a total of 80 insects tested per dual choice assay). The experiments were carried out under laboratory conditions (20 ± 1 °C, 65% ± 5% RH, and a 16L:8D photoperiod under cool white LED lights (77 µmoL/sqm/s)). These conditions were monitored using an automatic data logger (HOBO RH/TEMP 8 K; Onset Computer Corporation, Bourne, MA, USA).

### 2.4. Statistical Analyses

Binomial proportion tests (equal distribution hypothesized) were used to compare the number of males, females and eggs laid on each studied plant (the results of the four bioassay replicates were pooled together). All tests were performed using Minitab^®^ v.16 software [12].

## 3. Results

Table 1 lists the total numbers of males, females and eggs on each side of the tunnel. In terms of adult attraction, we found no preference for either level of infestations. Although nearly significant, we found no difference in terms of number of eggs laid on the non-infested plants* versus* the plants infested by 10 *T. absoluta* larvae (*p* = 0.086). In the dual choice including a non-infested plant* versus* a 20 larvae-infested tomato plant, females significantly preferred laying eggs on the non-infested plants (*p* = 0.047). Finally, females significantly preferred laying eggs on a 10 larvae-infested plant than on a 20 larvae-infested plant (*p* < 0.001).

**Table 1:** Results of dual choice bio-assays evaluating the preference of *T. absoluta* for non-infested, 10 larvae-infested and 20 larvae-infested tomato plants. The number of adult males and females, and eggs laid by females, was assessed in each zone/plant of the flying tunnel (0 = non-infested plant area, 10 = 10 larvae-infested plant area, 20 = 20 larvae-infested plant area). Values are total numbers of individuals for each combination (four replicates).

Number of Tested Insects	Responding Insects (%) ^1^		Two Choice Assay		*p*-value
81	72 (89)		10	0	
		Males	16	19	0.736
		Females	16	21	0.511
		Eggs	214	179	0.086
85	68 (80)		20	0	
		Males	12	23	0.090
		Females	16	17	1.000
		Eggs	163	202	0.047
82	55 (67)		20	10	
		Males	8	12	0.503
		Females	15	20	0.500
		Eggs	60	160	<0.001

^1^ Responding insects include living individuals present in one of the two side areas of the tunnel.

## 4. Discussion

This study shows that the oviposition site choice by female *T. absoluta* is likely to be dependent on the infestation level by conspecifics. It is generally assumed that larval survivability depends on the female’s oviposition choice because most lepidopteran larvae are unable to move to an alternative food source [13,14]. A key assumption could be that previous leafminer infestation will result in a less favorable host environment compared to an uninfested one as over-infested sites are unlikely to support growth and development of a newly laid egg. In the light of the results, high larvae density on tomato plants could be effectively associated by females with a poor quality host for egg laying. Indeed, the number of eggs laid on the plant infested by 20 *T. absoluta* larvae was lower than on plants with lower infestation levels in two tested combinations. However, female preferences are not restricted to the least infested plant and oviposition seems to be stimulated at a certain density threshold, as the number of eggs was found to be statistically similar between the non-infested plant and the plant infested with 10 larvae only (although the later was higher). This suggests that female oviposition response could be more sophisticated than would be expected from a simple intraspecific competition model based on the detection of previous infestation.

Considering *T. absoluta* antagonists may provide more explanations about this behavior. In their natural environment, solanaceous leafminers are significantly affected by many factors including natural enemies (as predators and parasitoids) that keep them at endemic levels [8]. Natural enemies were not involved in the present study but could be expected to play a role in forging the oviposition behavior of the tomato leafminer. Non-infested sites may pose some risks in terms of offspring survivability as early infesters may be exposed to predation or parasitism, while later infesters may have an increased chance to escape because at least partial satiation of the natural enemies has occurred [15]. Under these conditions, gravid leafminer females may have develop a system of host plant assessment manifested by moderate oviposition level on non-infested sites, increased level on sparsely infested sites, and reticent level on heavily infested sites.

During oviposition, females are exposed to a variety of cues, including plant volatiles, contact chemicals, and visual signals, which help to determine the suitability of a host plant [13]. By example, *T. absoluta* oviposition preferentially occurs on the upper third of the tomato plant where highest densities of trichomes (which impair parasites performance) can be found, resulting in clumped and patched leafminer distribution in tomato crops [16]. In the present experiment, we excluded the potential effect of the chemical host marking by ovipositing females on their preferences because the larvae used to infest the tomato plants in the experimental setup were excised from the rearing colony. However, many insect species mark the host patch during egg lay to regulate the oviposition site selection [17]. Especially, oviposition-deterring pheromones associated with egg lay are involved in reducing competition [18]. Moreover, egg deposition could be responsible for the release of oviposition-induced plant volatiles which could have an impact on female preferences [19]. Further researches can be oriented in studying the role of egg-infested plants in oviposition preferences by conspecific females.

We found differences in terms of total number of eggs laid on both plants, but no difference in terms of number of adult males and females in both sides of the tunnel. The total duration of the assay (48 h) and the design of the tunnel allow adults to freely move from one area to another, even after laying eggs on a plant. A shorter observation period (<1 h) would have probably allowed to determine the most attractive area for ovipositing females. *T. absoluta* females rely on olfactory cues during host-searching and host-assessment as suitable larval substrate for oviposition [10]. By contrast, lepidopteran males are primarily attracted by female sex pheromones and rarely exhibit adaptive behavior in response to host plant volatiles [20]. The lack of male preferences to plant combinations could also be related to the lack of female choice.

Polyphagous predators, as *Macrolophus pygmaeus* Rambur (Heteroptera: Miridae) which is a natural enemy of *T. absoluta*, are considered to be important candidate biological control agents [8]. Understanding the chemical volatiles involved both in *T. absoluta* oviposition choice and *M. pygmeus* attraction could help to develop reliable strategies in order to repulse gravid females from tomato crops and attract predators to infested sites, respectively. In an equivalent behavioral assay system, *M. pygmaeus* was previously found to discriminate a tomato plant infested by *T. absoluta* from a non-infested plant [21]. Volatile blends released by the tested plants were characterized and they were found to differ according to the infestation level (non-infested plants, plants infested by 10 larvae and 20 larvae). The volatile profiles were found to differ starting from 4 days after infestation. These authors also found that the more *T. absoluta* larvae are infesting the plant, the greater the amount of induced volatile chemicals released. As lepidopteran females are classically attracted to the odor blends released by plants which indicate mating sites and reflect larval host plant quality [11,22], we might expect that the volatile compounds responsible for the attraction of *M. pygmaeus* to infested tomato plants could also be involved in *T. absoluta* oviposition behavior. By contrast, egg deposition was not found to render tomato plants more attractive to leafminer predators as *M. pygmeus* [23], but oviposition-induced plant volatiles (if any) may also have an impact on gravid female behavior as explained above. Further electrophysiological and behavioral assays are required to confirm the effect of such specific chemicals on the choice of the oviposition site in *T. absoluta*.

## 5. Conclusions

This study suggests that *T. absoluta* oviposition behavior is dependent on the host plant infestation level by conspecifics. Preferences between conspecific-infested and non-infested host appear to be governed by a more complex mechanism than a simple intra-specific competition model, and other factors as parasites may have play a role in forging the leafminer oviposition behavior. These results provide useful information and perspectives for the development of further pest control strategies.
